# Ideal entry point and direction of retrograde intramedullary nailing of the tibia

**DOI:** 10.1186/s13018-023-03921-3

**Published:** 2023-06-29

**Authors:** Min He, Ziyu Jiang, Wenfu Tan, Zhengmao Li, Bin Peng

**Affiliations:** Department of Traumatic Orthopedics, Hengyang Medical School, The Second Affiliated Hospital, University of South China, Hengyang, Hunan 421001 China

**Keywords:** Tibia, Retrograde intramedullary nail, Point of entry, Entry direction, Surgery

## Abstract

**Purpose:**

To determine the ideal entry point and direction of retrograde intramedullary nailing of the tibia.

**Methods:**

The imaging data of patients with distal tibial fractures from June 2020 to December 2021 in our hospital were collected, and computer-aided design was performed. The relevant data were imported into the software for processing, so as to obtain a distal tibial fracture model and simulate the retrograde intramedullary nail placement in the tibia. The entry points and angles at which the intramedullary nail could be inserted successfully and the fracture could be maintained in good alignment were overlapped and counted to obtain the safe entry range and angle. The center of this safe range is the ideal entry point for retrograde intramedullary nailing of the tibia, and the mean value of the angle is the ideal direction of entry.

**Results:**

The ideal entry point of the retrograde intramedullary nailing was located at the midpoint of the medial malleolus in the C-arm fluoroscopic anteroposterior (AP) and lateral view. The ideal nail entry direction was located at the anatomic axis of the medial malleolus in the AP position and at the anatomic axis of the distal tibial metaphysis in the lateral position.

**Conclusion:**

The ideal point and direction of nail insertion for retrograde tibial intramedullary nailing is a "double midpoint, double axis" approach.

## Introduction

Distal tibial fractures are one of the common types of fractures in clinical practice. One study showed that the annual incidence of distal tibial fractures is about 9.1 / 100,000, accounting for 18.3% of all tibial fractures [1]. Distal tibial fractures are unique in that they are prone to complications such as infection, poor fracture repositioning, delayed fracture healing and nonunion, making them difficult to treat clinically [2, 3]. Currently, anterograde tibial intramedullary nailing and plate are the two main internal fixation modalities, but both have certain advantages and disadvantages that need to be considered in the surgical decision [4–6]. In recent years, a novel retrograde tibial intramedullary nail (RTN) has been designed to provide a new option for the minimally invasive treatment of distal tibial fractures [7]. However, there are few relevant studies on RTN, especially the ideal entry point and direction of the nail is not well defined. In this study, we propose to apply computer-aided design to determine the ideal entry point and direction by constructing a distal tibial fracture model and simulating RTN placement, so as to provide a more solid basis for the clinical application of this technique.

## Materials and methods

Imaging data of patients with distal tibial fractures from June 2020 to December 2021 were collected from our hospital. Among them, distal tibial fracture was defined as a fracture in which the fracture line was located within 11 cm of the ankle joint surface, according to the AO/OTA fracture classification: 43-A1/A2/A3, 43-C1/C2. In addition, patients had bilateral CT scans of the ankle joint, in which one side was a distal tibial fracture and the other side was normal. We collected a total of 26 patients with distal tibial fractures meeting the above criteria, and the obtained imaging data were stored in DICOM format. Informed consent was obtained from all patients and ethical committee approval was obtained.

**Fig. 1:**
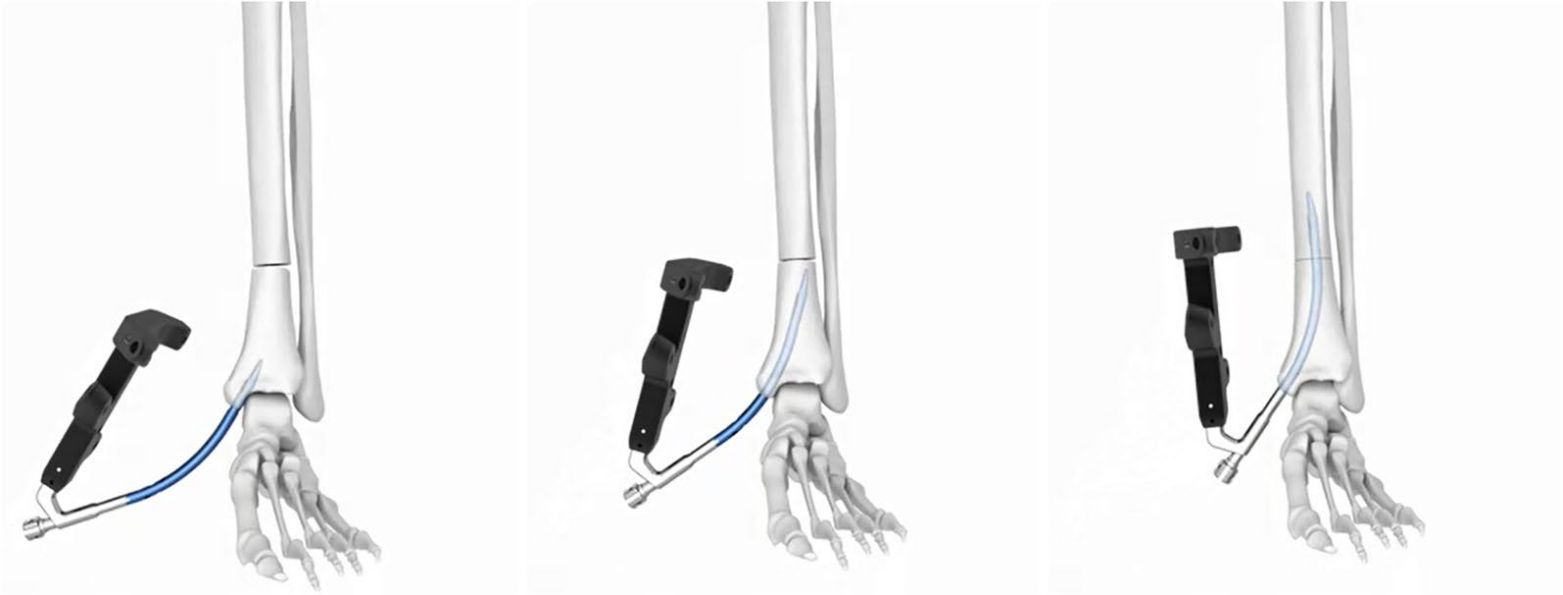
Simulation placement of RTN

**Fig. 2:**
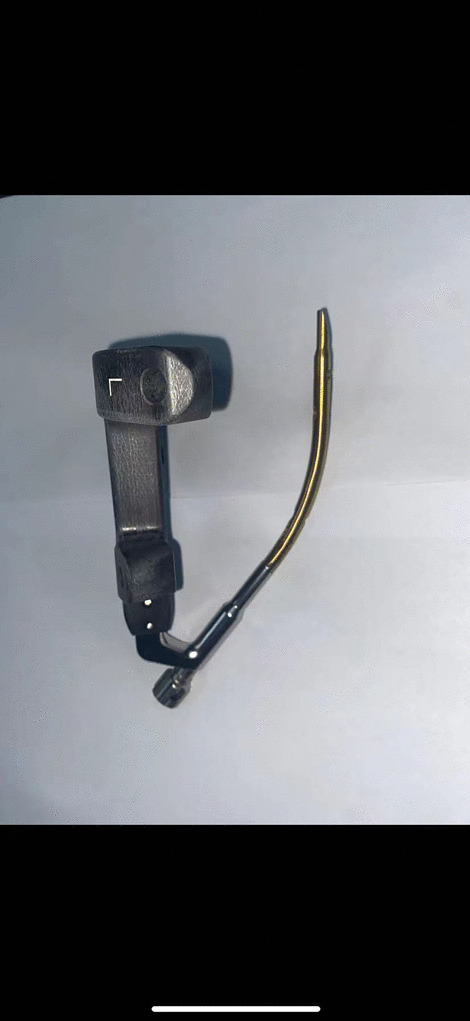
The figure of RTN

We measured the length of the distal tibial fracture line from the ankle joint surface in 26 patients and obtained an average value of 8.5 cm. The imaging data of the tibia on the normal side of the patient were imported into Mimics software and the preliminary 3D point cloud model of the tibia was constructed by 3D reconstruction and saved in STL format. The above data were processed by Geomagic Studio software to obtain the Nurbs surface, which was saved in IGS format. Finally, in Solidworks software, the transverse fracture line was simulated and drawn at a distance of 8.5 cm from the distal tibial plafond, and the fracture was set to an angle of 5° in the coronal plane and 10° in the sagittal plane. The placement of the RTN was simulated on the above distal tibial fracture model (Fig. 1). The entry points and angles at which the intramedullary nail could be inserted smoothly and the fracture could be maintained in good alignment were overlapped and counted to obtain the safe entry range and angle of the RNT. The center of this safe range is the ideal entry point of the RTN, and the mean value of the angle is the ideal direction of entry. The internal fixation model is based on the data of RTN from Double Medical Technology Inc(China). We obtained the data related to the 8.0 mm*140 mm size RTN (Fig. 2) by laser scanning, saved it in stl format, and imported the above data into Solidworks software for RTN modeling.

## Results

**Fig. 3:**
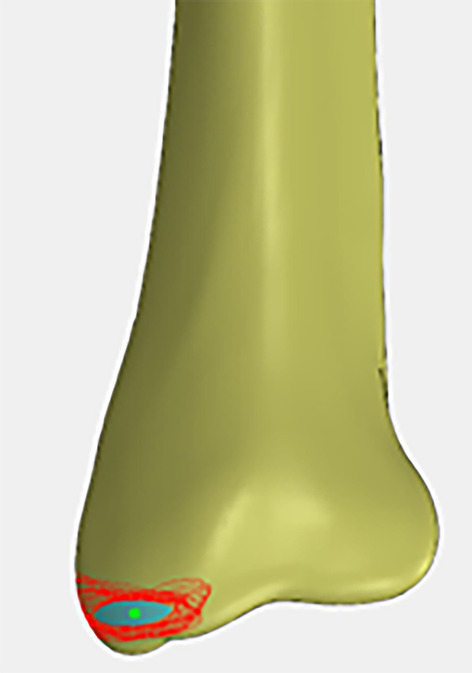
The safe range of RNT insertion and the ideal entry point

**Fig. 4:**
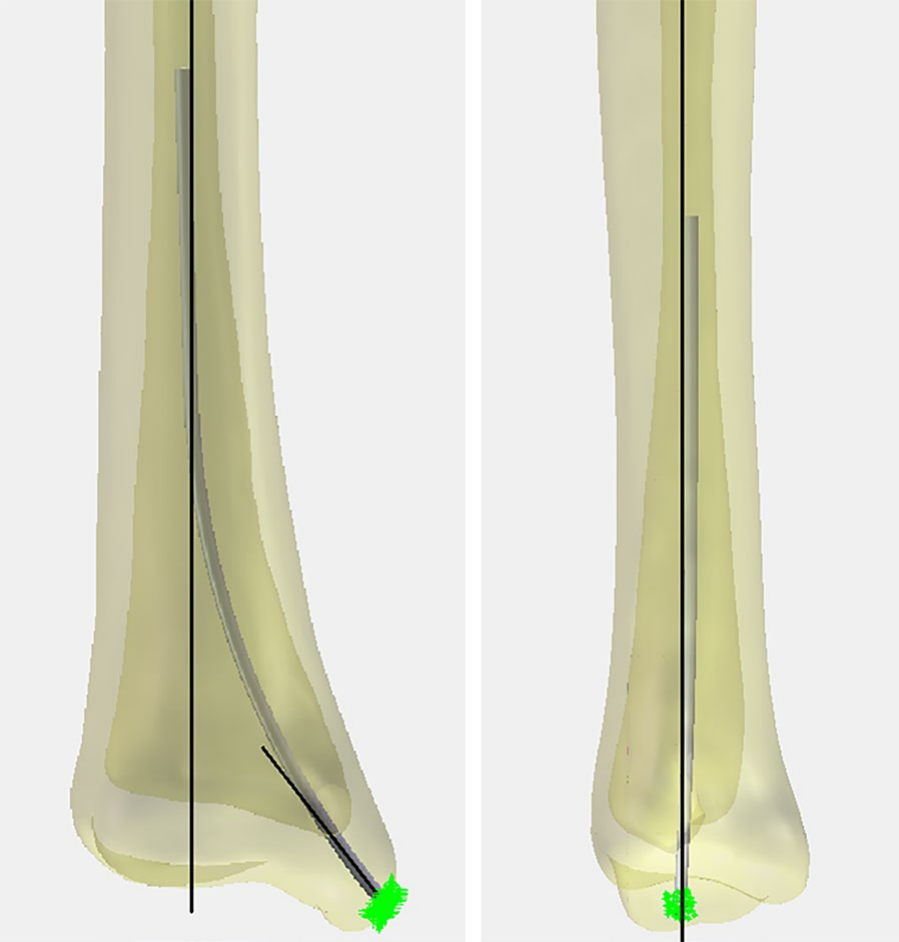
The ideal entry angular of the RTN

**Fig. 5:**
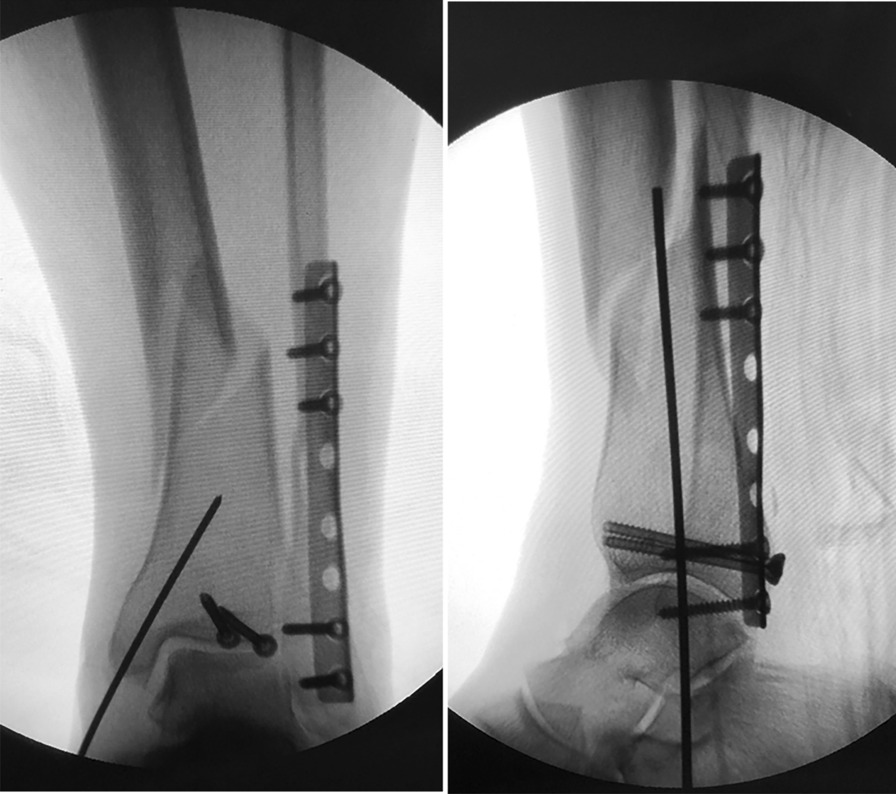
The “double midpoint, double axis” approach of RTN in clinical

The safe range of RNT insertion was in the shape of a shuttle, with an area of about 31.6 mm^2^. The highest point was 6.0 mm from the vertical distance of the horizontal tangent line of the intercollicular groove apex of the medial malleolus, and the lowest point was 2.9 mm., The most anterior point was 6.6 mm from the horizontal distance of the vertical tangent line of the medial malleolus front, and the last point was 4.0 mm from the horizontal distance of the vertical tangent line of the medial malleolus posterior. The center point of this range is in the position of the superior edge of the intercollicular groove apex of the medial malleolus, which locates at the midpoint of the line between the anterior and posterior medial malleolus margins at this level (Fig. 3). With the anatomic axis of the distal tibia as a reference, the safe angular range of the RTN was 40.0° ± 1.7° in the coronal position, and the mean value of this angle is basically at the anatomic axis of the medial malleolus. Meanwhile, the safe angular is at the anatomic axis of the distal tibial metaphysis in the lateral position (Fig. 4). In addition, during clinical manipulation, we found that the superior edge of the intercollicular groove apex was essentially at the midpoint of the medial and lateral cortices of the medial malleolus with the C-arm fluoroscopy in the AP position. In summary, the ideal entry point for RTN is located at the midpoint of the medial malleolus in both the AP and lateral positions, and the ideal direction of entry is at the anatomic axis of the medial malleolus in the AP position and at the anatomic axis of the distal tibial metaphysis in the lateral position (Fig. 5).

## Discussion

The intramedullary nailing system is one of the major internal fixation modalities for extremity fractures. With the innovation of design and technology, the application of intramedullary nailing has become more and more widespread [8]. Currently, the new RNT has been clinically used in the treatment of distal tibial fractures. Despite the many advantages of this intramedullary nailing system, there are some controversies that need to be further explored, such as the ideal point and direction of nail insertion. In the few articles available, the description of the entry point and direction of the RTN is not very clear. Some articles simply state that the nail is introduced through the medial cortex near the tip of the medial malleolus [7, 9, 10]. while Sebastian Kuhn further states that the wire was drilled parallel to the medial cortex at a distance of approximately 5 mm [11]. However, it lacks a description of the lateral position and does not state the basis for the selection of this entry point and the direction of the nail. In addition, the C-arm fluoroscopy does not directly show the "5 mm" distance, which makes it difficult to accurately locate it intraoperatively.

The entry point and direction of intramedullary nailing are very important, and improper selection may lead to a series of complications, such as poor fracture repositioning and medically induced injuries [12–14]. Therefore, it is necessary to clarify the ideal entry point and direction of RTN for its clinical application. In recent years, Computer Aided Design (CAD) has been widely used in the field of orthopedics [15–17]. In this study, we collected imaging data from patients with distal tibial fractures and performed CAD, which led to the desired distal tibial fracture model. First, by collecting data from 26 patients with distal tibial fractures, we determined the mean value of the fracture line length from the ankle joint surface to be 8.5 cm. Subsequently, we simulated drawing the transverse fracture line at a distance of 8.5 cm from the tibial spur joint surface using the tibia model on the normal side of the patient as the study subject. Lastly, the fractures were set at an angle of 5° in the coronal plane and 10° in the sagittal plane, which are the critical reduction criteria for tibial fractures [18]. The placement of RTN was simulated on this distal tibial fracture model, and if the intramedullary nail could be successfully placed and maintain good reduction in the fracture, it indicated that the entry point and direction were appropriate. On the contrary, if the intramedullary nail could not be inserted or the fracture end was displaced, the point and direction were inappropriate. We overlapped the appropriate entry points and counted the entry angles to obtain the safe entry range and angle of RNT.

Through CAD and clinical experience, we believe that the ideal entry point of RTN is located at the midpoint of the medial malleolus in the AP and lateral position of C-arm fluoroscopic. The ideal entry direction is orthogonally at the anatomical axis of the medial malleolus and laterally at the anatomical axis of distal tibial metaphysis. Compared with the previous studies, we believe that the above "double midpoint, double axis" approach has some advantages. First, the "midpoint of the medial malleolus" is more intuitive and more maneuverable than "a distance of approximately 5 mm from medial malleolus" in C-arm fluoroscopy, which may help to reduce fluoroscopies and shorten the operative time. In addition, we further determined the ideal entry point and direction of RTN in the C-arm fluoroscopic lateral position compared with the previous literature. Meanwhile, the "double midpoint, double axis" approach minimizes the risk of penetration of the intramedullary nail into the medial malleolus cortex. It has been shown that the thickness of the medial malleolus is only about 12 mm [19], and the diameter of the RTN is 8–10 mm. If the nail entry point is too far from the midpoint, which will increase the risk of medial malleolus injury. The distal tibial marrow cavity gradually widens, and this special structure allows for a larger space for the entry of the intramedullary nail. The "double midpoint, double axis" approach allows RTN to fit better into the distal tibial marrow cavity, which facilitates fracture reduction and fixation. At present, we have used this entry point and direction for RTN insertion in clinical, achieving a good efficacy in the treatment of distal tibial fractures (20).

## Conclusion

In conclusion, the ideal point and direction of nail insertion for retrograde tibial intramedullary nailing is a "double midpoint, double axis" approach. However, there are certain shortcomings in this study. The CAD were determined based on the parameter of RTN from Double Medical Technology Inc, but there may be some differences between different companies. In addition, the effect of this entry point and direction on the medial malleolus is not very clear, and its adjacent relationship with the tendon, vascular nerves and ligaments around the medial malleolus needs further study.

## Data Availability

Not applicable.
